# Supplementary material for: Protective effect of TCR-mediated MAIT cell activation during experimental autoimmune encephalomyelitis
Source: Nat Commun. 2024 Oct 28;15:9287. doi: 10.1038/s41467-024-53657-9 (PMC11519641; doi:10.1038/s41467-024-53657-9)
Supplement: Supplementary file 1 — Supplementary information [file 41467_2024_53657_MOESM1_ESM.pdf]

Protective effect of TCR-mediated MAIT cell activation during experimental autoimmune encephalomyelitis

AUTHORS

Mark Walkenhorst[1], Jana K. Sonner[1], Nina Meurs[1], Jan Broder Engler[1], Simone Bauer[1], Ingo Winschel[1], Marcel S. Woo[1], Lukas Raich[1], Iris Winkler[1], Vanessa Vieira[1], Lisa Unger[1], Gabriela Salinas[2], Olivier Lantz[3], Manuel A. Friese[1,*,†], Anne Willing[1,*,†]

AFFILIATIONS

[1]Institute of Neuroimmunology and Multiple Sclerosis, University Medical Center Hamburg-Eppendorf, Hamburg, Germany.

[2]NGS-Integrative Genomics Core Unit, Institute of Pathology, University Medical Center Göttingen, Göttingen, Germany.

[3]Institut National de la Santé et de la Recherche Médicale U932, PSL University, Institut Curie, Paris, France.

*These authors contributed equally: Manuel A. Friese, Anne Willing

†Corresponding authors: Manuel A. Friese (manuel.friese@zmnh.uni-hamburg.de) and Anne Willing (anne.willing@zmnh.uni-hamburg.de)

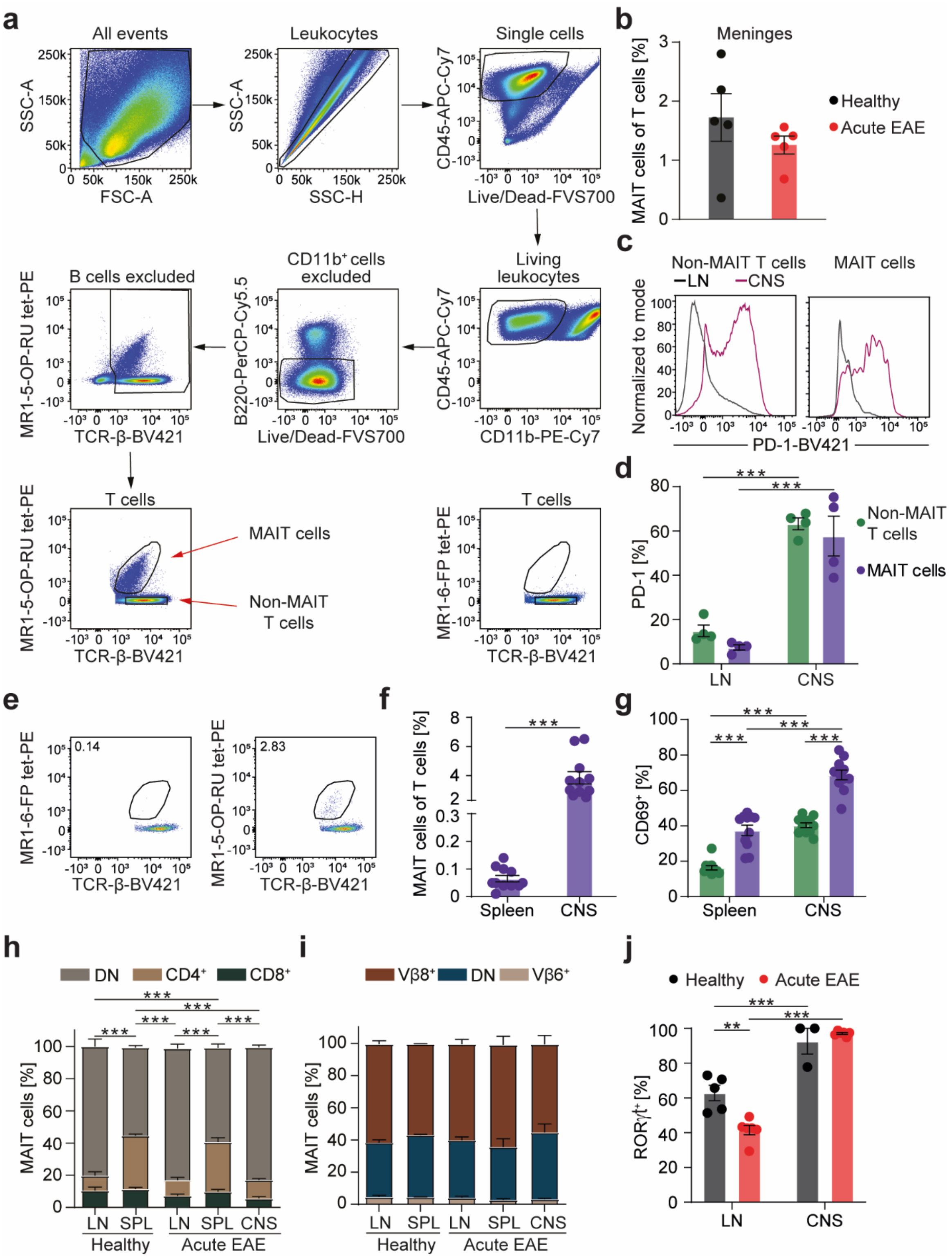

Suppl. Fig. 1: Characterization of MAIT cells in the EAE model. (**a**) Gating strategy for MAIT cells among immune cells isolated from the inflamed CNS identified as living CD45$^+$CD11b$^-$CD45R$^-$TCR-β$^+$MR1tetramer(5-OP-RU)$^+$ cells. (**b**) MAIT cell frequency in dural meninges of healthy C57BL/6J mice (*n* = 5) and C57BL/6J mice in acute EAE (15 days post immunization (dpi)) (*n* = 5) was quantified by flow cytometry. (**c, d**) PD-1 expression of non-MAIT T cells (living CD45$^+$CD11b$^-$CD45R$^-$TCR-β$^+$MR1tetramer(5-OP-RU)$^-$CD44$^+$ cells; *n* = 4) and MAIT cells (*n* = 4) in LN and CNS of C57BL/6J mice during acute EAE (14 dpi) was analyzed by flow cytometry. (**e-g**) EAE was induced by adoptive transfer of MOG$_{35-55}$ autoreactive T cells in C57BL/6J mice (*n* = 11). (**e, f**) MAIT cell frequency in spleen and CNS

during acute EAE (12–15 days post transfer). (**g**) T cell activation, reflected by CD69 expression, was quantified by flow cytometry in spleen and CNS during acute EAE (12–15 days post transfer). (**h**) MAIT cells from healthy C57BL/6J mice (*n* = 5) and C57BL/6J mice during acute EAE (14 dpi; LN and spleen, *n* = 5; CNS, *n* =4) were analyzed by flow cytometry and classified as $CD4^+$, $CD8^+$ and double-negative (DN). (**i**) MAIT cells from healthy C57BL/6J mice (LN, *n* = 7; Spleen, *n* = 2) and C57BL/6J mice during acute EAE (14 dpi) (LN and CNS, *n* = 5; Spleen, *n* =3) were analyzed by flow cytometry and classified as TCR-Vβ6^+, TCR-Vβ8^+ and double-negative (DN). (**j**) RORγt expression in CNS (*n* = 3) and LN (*n* = 5) from healthy RORγtGFP transgenic reporter mice and during acute EAE (*n* = 6 per group) was quantified by flow cytometry. Data are shown as mean ± SEM. Statistics: t-test (two-tailed) in **b**, **f** (P = 0.0004); one-way ANOVA on frequency of $CD4^+$ or TCR-Vβ8^+ cells respectively in **h**, **i**; two-way ANOVA in **d**, **g, j**; $^{**}P < 0.01$, $^{***}P < 0.001$. Source data are provided as a Source Data file.

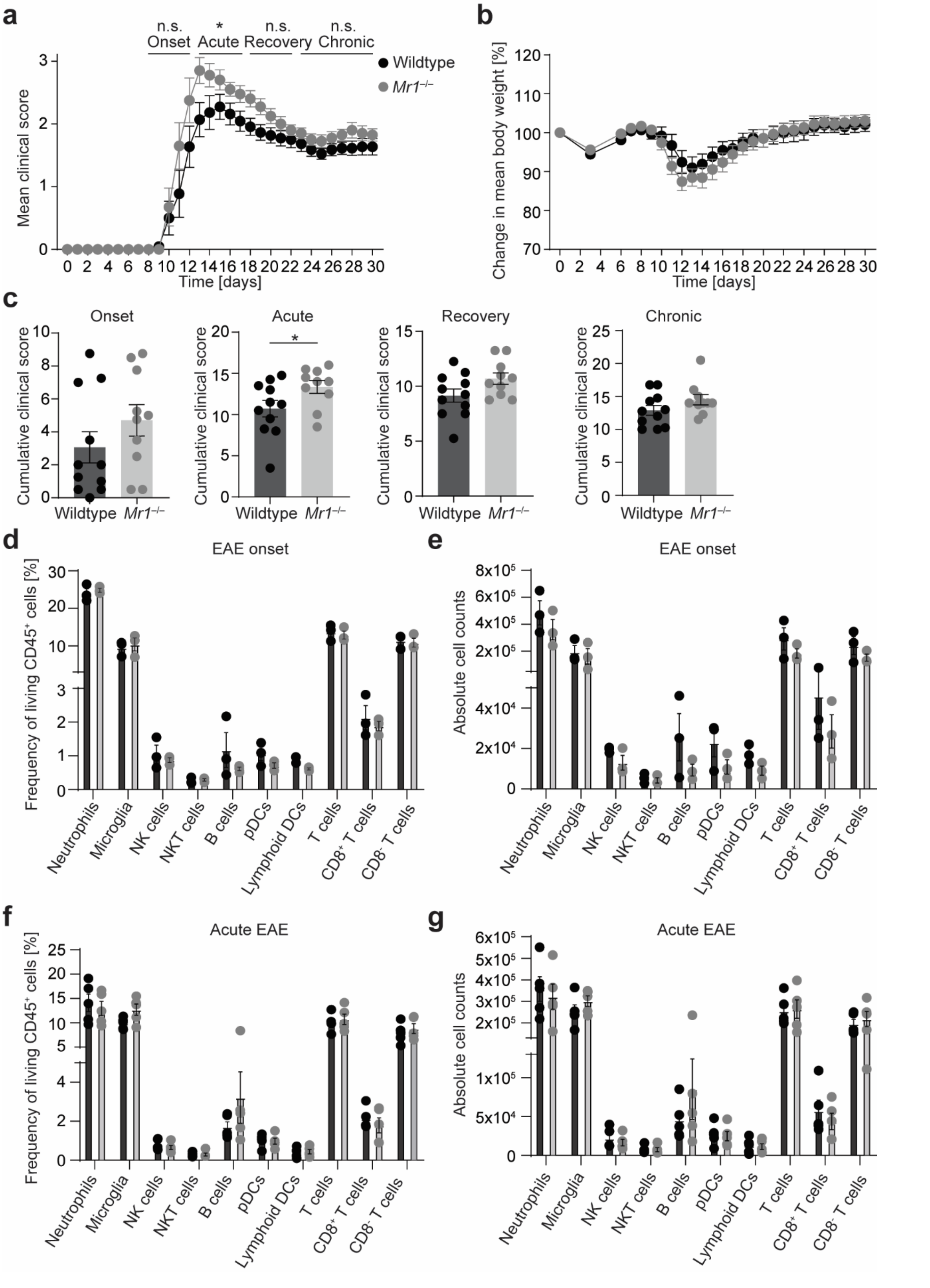

Suppl. Fig. 2: Exacerbated EAE course of *Mr1*$^{–/–}$ mice with unaltered immune cell infiltration in the CNS. (**a-c**) EAE was induced in *Mr1*$^{–/–}$ mice (*n* = 10) and wildtype littermates (*n* = 11) by active immunization against $MOG_{35\text{-}55}$. Clinical score and body weight were assessed daily. The EAE course was divided into EAE onset (day of first symptoms–12 days post immunization (dpi)), acute EAE (13–17 dpi), EAE recovery (18–22 dpi) and chronic EAE

(23–30 dpi). The cumulative score during the respective phases was compared between groups. (**d, e**) CNS immune cell phenotyping was performed from *Mr1*$^{-/-}$ mice (*n* = 3) and wildtype littermates (*n* = 3) at EAE onset (10 dpi). Frequencies and absolute numbers of immune cell populations were analyzed by flow cytometry. (**f, g**) CNS immune cell phenotyping was performed from *Mr1*$^{-/-}$ mice (*n* = 5) and wildtype littermates (*n* = 5) during acute EAE (14 dpi). Frequencies and absolute numbers of immune cell populations were analyzed by flow cytometry. Data are shown as mean ± SEM. Statistics: Mann-Whitney-U test (two-tailed) in **a**, **c** (P = 0.0491); two-way ANOVA in **d**, **e**, **f**, **g**; *P < 0.05. Source data are provided as a Source Data file.

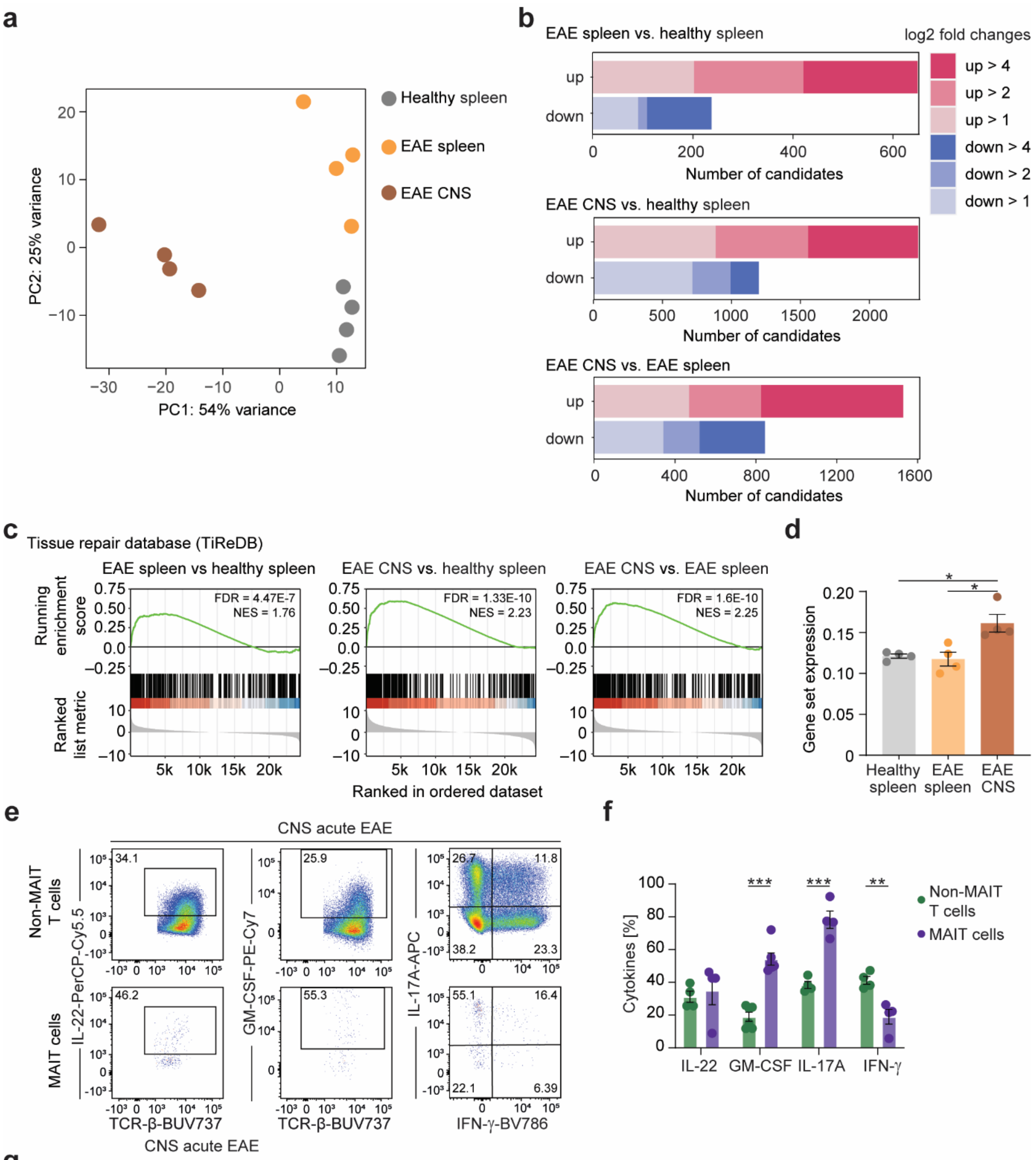

g

Top 10 leading edge genes of the commensal-specific CD8⁺ T cell tissue repair GSEA

	EAE spleen vs. healthy spleen	EAE CNS vs. healthy spleen	EAE CNS vs. EAE spleen
1	*Thbs1*	*Tnf*	*Cxcl1*
2	*Csf1*	*Cxcl1*	*Tnf*
3	*Il1b*	*Csf2*	*Hif1a*
4	*Vegfa*	*Cxcl10*	*Cxcl10*
5	*Tgfb1*	*Areg*	*Csf2*
6	*Ccl3*	*Hif1a*	*Pdgfa*
7	*Nrg2*	*Furin*	*Tgfb3*
8	*Wnt10a*	*Csf1*	*Furin*
9	*Areg*	*Tgfb1*	*Areg*
10	*Vegfb*	*Thbs1*	*Ccl2*

Suppl. Fig. 3: Transcriptome profiling of MAIT cells in EAE. (**a**) Principal component analysis (PCA) of RNA sequencing data of MAIT cells from the spleen of healthy mice and mice during acute EAE (14 days post immunization (dpi)) as well as from the CNS during acute EAE (*n* = 4 samples per group pooled from 5 mice per sample). (**b**) Number of differentially

expressed genes in indicated comparisons. Color code represents log2 fold changes for up- and downregulated genes. (**c**) Gene set enrichment analysis (GSEA) of a signature defining tissue repair from a public database (TiReDB)[34]. Normalized Enrichment Score (NES) and false discovery rate (FDR; *P* values after Benjamini-Hochberg adjustment) are shown. (**d**) Quantification of expression of TiReDB tissue repair signature in MAIT cells derived from healthy spleen, EAE spleen and EAE CNS by AUCell analysis (*n* = 4 samples per group pooled from 5 mice per sample). (**e**, **f**) Lymphocytes were isolated from the inflamed CNS of C57BL/6J mice during acute EAE (14 dpi) and were cultured for 4 hours in the presence of phorbol 12-myristate-13-acetate (PMA) (10 ng/ml), ionomycin (1 µg/ml) and monensin (2 µM). IL-22 (*n* = 4 mice), GM-CSF (*n* = 6 mice), IL-17A (*n* = 4 mice) and IFN-γ (*n* = 4 mice) were subsequently stained intracellularly and quantified by flow cytometry. (**g**) Top ten leading edge genes of GSEA from **Fig. 2d**. *Amphiregulin* (*Areg*) is marked in red. Bars represent mean ± SEM. Statistics: gene set enrichment analysis (GSEA) in **c**; one-way ANOVA in **d**; two-way ANOVA in **f**; *$P < 0.05$, **$P < 0.01$, ***$P < 0.001$. Source data are provided as a Source Data file.

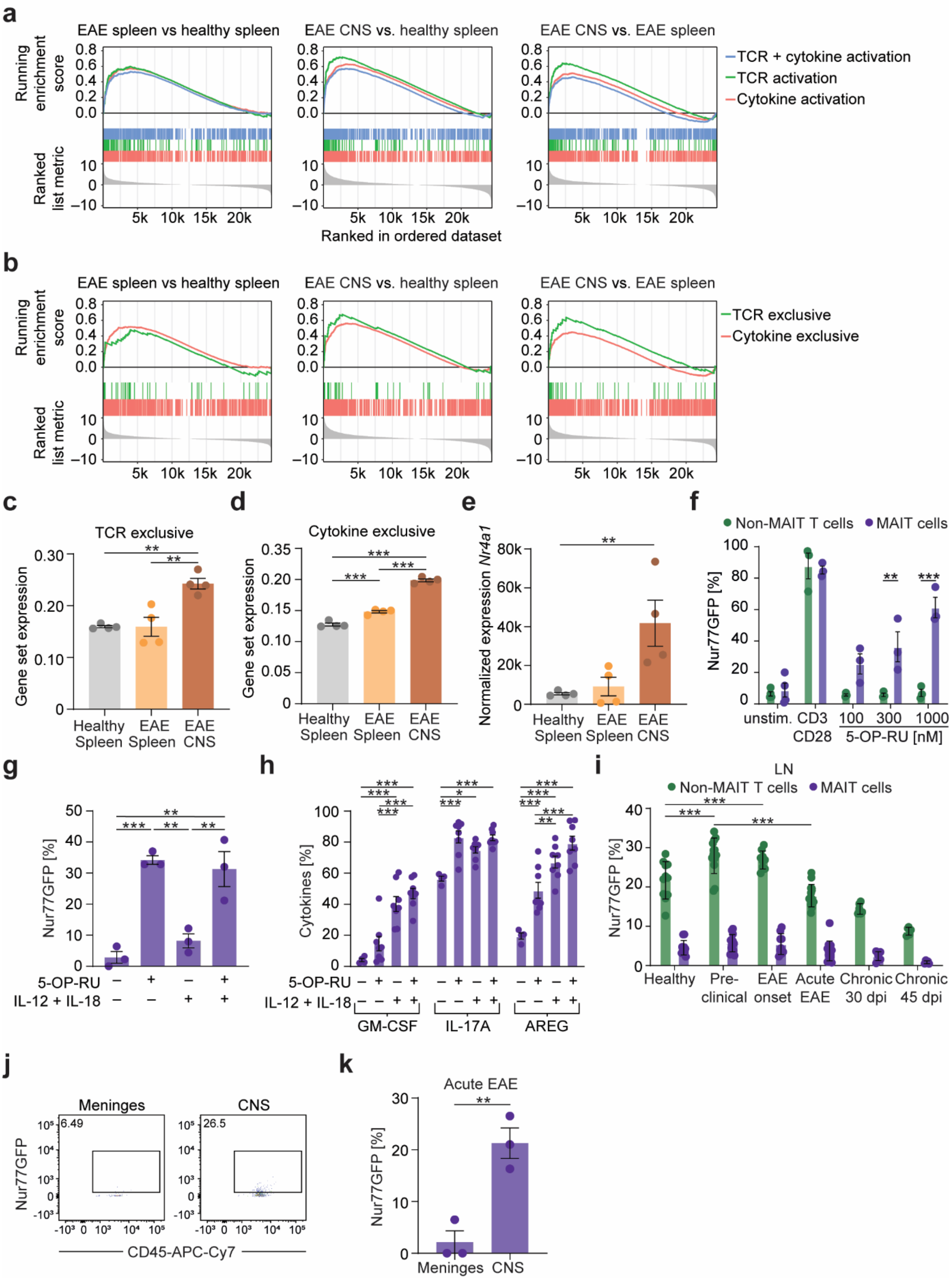

Suppl. Fig. 4: TCR- and cytokine-mediated activation of MAIT cells in EAE. (**a**) Gene set enrichment analysis (GSEA) of signatures defining MAIT cells activated via cytokines, TCR or via cytokines and TCR[19] in indicated comparisons. (**b**) TCR exclusive and cytokine exclusive gene lists were generated by removing the overlap between the TCR activation and cytokine activation gene sets[22]. GSEA of TCR exclusive and cytokine exclusive signatures are shown in indicated comparisons. (**c**, **d**) Expression of gene sets from TCR exclusive and cytokine exclusive signatures in sorted MAIT cells from indicated tissues

quantified by AUCell analysis (*n* = 4 samples per group pooled from 5 mice per sample). (**e**) Normalized expression of *Nr4a1* (Nur77) from RNA sequencing data of sorted MAIT cells (*n* = 4 samples per group pooled from 5 mice per sample). (**f**) Lymphocytes from the liver of Nur77GFP reporter mice were isolated and cultured for 3 days in the presence of 5-OP-RU at indicated concentrations or anti-CD3 (clone: 145-2C11, 0.125 µg/ml) and anti-CD28 (clone: 37.51, 0.25 µg/ml). After 2 days 5-OP-RU was added again in the respective concentration. Nur77GFP expression of non-MAIT T cells and MAIT cells was analyzed by flow cytometry (unstimulated, *n* = 4 mice; activation conditions, *n* = 3 mice). Data from three independent experiments. (**g**) Lymphocytes from the liver of Nur77GFP reporter mice (*n* = 3) were isolated and cultured for 2 days in the presence of 5-OP-RU (500 nM) and/or IL-12 (10 ng/ml) and IL-18 (12.5 ng/ml) or left unstimulated. Nur77GFP expression was quantified by flow cytometry. (**h**) Lymphocytes from the liver were isolated and cultured as in **g**. After 2 days, phorbol 12-myristate-13-acetate (PMA) (10 ng/ml), ionomycin (1 µg/ml) and monensin (2 µM) were added for 4 hours. Intracellular GM-CSF, IL-17A and AREG were quantified by flow cytometry (unstimulated, *n* = 4 mice; activation conditions, *n* = 8 mice). (**i**) Nur77 expression of non-MAIT and MAIT cells in LN was quantified by flow cytometry over the course of EAE and in healthy Nur77GFP reporter mice. Preclinical EAE (6–7 days post immunization (dpi); *n* = 13), EAE onset (9–11 dpi; *n* = 9), acute EAE (13-14 dpi; *n* = 14) and chronic EAE (30 dpi, *n* = 6; 45 dpi, *n* = 5). (**j**, **k**) Nur77GFP expression of MAIT cells from dural meninges and CNS from Nur77GFP reporter mice (*n* = 3) in acute EAE (15 dpi) was analyzed by flow cytometry. Data are shown as mean ± SEM. Statistics: gene set enrichment analysis (GSEA) in **a**, **b**; one-way ANOVA in **c**, **d**; DESeq2 false discovery rate-adjusted P value in **e** (*P* = 0. 0046); two-way ANOVA in **f**, **g**, **h**, **i**; t-test (two-tailed) in **k** (*P* = 0. 0064); **P* < 0.05, ***P* < 0.01, ****P* < 0.001. Source data are provided as a Source Data file.
