# Supplementary material for: Protective effect of TCR-mediated MAIT cell activation during experimental autoimmune encephalomyelitis
Source: Nat Commun. 2024 Oct 28;15:9287. doi: 10.1038/s41467-024-53657-9 (PMC11519641; doi:10.1038/s41467-024-53657-9)
Supplement: Supplementary file 2 — Description of Additional Supplementary Files [file 41467_2024_53657_MOESM2_ESM.pdf]

Description of Additional Supplementary Data

File Name: Supplementary Data 1

Description: Excel table of differential gene expression analysis of sorted MAIT cells from healthy spleen, EAE spleen and EAE CNS during acute EAE (14 days post immunization). The table includes the comparisons EAE CNS versus EAE spleen, EAE CNS versus heathy spleen and EAE spleen versus healthy spleen (n = 4 samples per group, 5 animals pooled per sample). Statistical analysis was performed using DESeq2 (v.1.36.0) defining genes with a minimal twofold change and false discovery rate (FDR)-adjusted $P < 0.05$ differentially expressed.

File Name: Supplementary Data 2

Description: Excel table of biological process (BP) gene ontology (GO) analysis of sorted MAIT cells from healthy spleen, EAE spleen and EAE CNS during acute EAE (14 days post immunization). The table includes the comparisons EAE CNS versus EAE spleen, EAE CNS versus heathy spleen and EAE spleen versus healthy spleen (n = 4 samples per group, 5 animals pooled per sample). Statistical analysis was performed by gene set enrichment analysis using the R package clusterProfiler (v4.4.4).

File Name: Supplementary Data 3

Description: Excel table of custom gene sets derived from the literature, including MAIT cell activation (Leng *et al*., 2019), tissue repair (Linehan *et al*., 2018; Yanai *et al*., 2016) and pathogenic Th17 cells (Lee *et al*., 2012). TCR exclusive and cytokine exclusive gene sets were generated by removing the overlap between the TCR activation and cytokine activation gene sets from Leng *et al*., 2019.

File Name: Supplementary Data 4

Description: Excel table of enrichment analysis using custom gene sets (Supplementary Data 3) against sorted MAIT cells from healthy spleen, EAE spleen and EAE CNS during acute EAE (14 days post immunization). The table includes the comparisons EAE CNS versus EAE spleen, EAE CNS versus heathy spleen and EAE spleen versus healthy spleen (n = 4 samples per group, 5 animals pooled per sample). Statistical analysis was performed by gene set enrichment analysis using the R package clusterProfiler (v4.4.4).

File Name: Supplementary Data 5

Description: Excel table of AUCell analysis using custom gene sets (Supplementary Data 3) against sorted MAIT cells from healthy spleen, EAE spleen and EAE CNS during acute EAE (14 days post immunization). The table includes the comparisons EAE CNS versus EAE

spleen, EAE CNS versus heathy spleen and EAE spleen versus healthy spleen (n = 4 samples per group, 5 animals pooled per sample). Analysis was performed using the R package AUCell (v.1.18.1).
