# Supplementary material for: Protective effect of TCR-mediated MAIT cell activation during experimental autoimmune encephalomyelitis
Source: Nat Commun. 2024 Oct 28;15:9287. doi: 10.1038/s41467-024-53657-9 (PMC11519641; doi:10.1038/s41467-024-53657-9)
Supplement: Supplementary file 8 — Reporting Summary [file 41467_2024_53657_MOESM8_ESM.pdf]

nature portfolio

Corresponding author(s): Manuel A. Friese
Anne Willing

Last updated by author(s): Oct 8, 2024

Reporting Summary

Nature Portfolio wishes to improve the reproducibility of the work that we publish. This form provides structure for consistency and transparency in reporting. For further information on Nature Portfolio policies, see our Editorial Policies and the Editorial Policy Checklist.

Statistics

For all statistical analyses, confirm that the following items are present in the figure legend, table legend, main text, or Methods section.

n/a	Confirmed	
☐	☒	The exact sample size (*n*) for each experimental group/condition, given as a discrete number and unit of measurement
☐	☒	A statement on whether measurements were taken from distinct samples or whether the same sample was measured repeatedly
☐	☒	The statistical test(s) used AND whether they are one- or two-sided<br>*Only common tests should be described solely by name; describe more complex techniques in the Methods section.*
☒	☐	A description of all covariates tested
☐	☒	A description of any assumptions or corrections, such as tests of normality and adjustment for multiple comparisons
☐	☒	A full description of the statistical parameters including central tendency (e.g. means) or other basic estimates (e.g. regression coefficient) AND variation (e.g. standard deviation) or associated estimates of uncertainty (e.g. confidence intervals)
☐	☒	For null hypothesis testing, the test statistic (e.g. *F*, *t*, *r*) with confidence intervals, effect sizes, degrees of freedom and *P* value noted<br>*Give P values as exact values whenever suitable.*
☒	☐	For Bayesian analysis, information on the choice of priors and Markov chain Monte Carlo settings
☒	☐	For hierarchical and complex designs, identification of the appropriate level for tests and full reporting of outcomes
☒	☐	Estimates of effect sizes (e.g. Cohen's *d*, Pearson's *r*), indicating how they were calculated

Our web collection on statistics for biologists contains articles on many of the points above.

Software and code

Policy information about availability of computer code

Data collection	Flow cytometry data were collected with FACSDiva software version 9.1 (BD Biosciences), HiSeq 4000 sequencer (Illumina)
Data analysis	GraphPad Prism software version 9.3.1, FlowJo analysis software version 10.8, Fiji (ImageJ, version 2.0.0), aligner STAR (version 2.5) with default settings, featureCounts, R environment (v.4.2.3), DESeq2 (v.1.36.0), R package clusterProfiler (v4.4.4), AUCell (v.1.18.1)

For manuscripts utilizing custom algorithms or software that are central to the research but not yet described in published literature, software must be made available to editors and reviewers. We strongly encourage code deposition in a community repository (e.g. GitHub). See the Nature Portfolio guidelines for submitting code & software for further information.

Data

Policy information about availability of data

All manuscripts must include a data availability statement. This statement should provide the following information, where applicable:

- Accession codes, unique identifiers, or web links for publicly available datasets
- A description of any restrictions on data availability
- For clinical datasets or third party data, please ensure that the statement adheres to our policy

Data generated for this study are available through the Gene Expression Omnibus under accession number GSE234291 (https://www.ncbi.nlm.nih.gov/geo/query/acc.cgi?acc=GSE234291). All other data are included in the Supplementary Information/ Data and Source Data file. Source data are provided with this paper.

Research involving human participants, their data, or biological material

Policy information about studies with human participants or human data. See also policy information about sex, gender (identity/presentation), and sexual orientation and race, ethnicity and racism.

Reporting on sex and gender	*Use the terms sex (biological attribute) and gender (shaped by social and cultural circumstances) carefully in order to avoid confusing both terms. Indicate if findings apply to only one sex or gender; describe whether sex and gender were considered in study design; whether sex and/or gender was determined based on self-reporting or assigned and methods used. Provide in the source data disaggregated sex and gender data, where this information has been collected, and if consent has been obtained for sharing of individual-level data; provide overall numbers in this Reporting Summary. Please state if this information has not been collected. Report sex- and gender-based analyses where performed, justify reasons for lack of sex- and gender-based analysis.*
Reporting on race, ethnicity, or other socially relevant groupings	*Please specify the socially constructed or socially relevant categorization variable(s) used in your manuscript and explain why they were used. Please note that such variables should not be used as proxies for other socially constructed/relevant variables (for example, race or ethnicity should not be used as a proxy for socioeconomic status). Provide clear definitions of the relevant terms used, how they were provided (by the participants/respondents, the researchers, or third parties), and the method(s) used to classify people into the different categories (e.g. self-report, census or administrative data, social media data, etc.) Please provide details about how you controlled for confounding variables in your analyses.*
Population characteristics	*Describe the covariate-relevant population characteristics of the human research participants (e.g. age, genotypic information, past and current diagnosis and treatment categories). If you filled out the behavioural & social sciences study design questions and have nothing to add here, write "See above."*
Recruitment	*Describe how participants were recruited. Outline any potential self-selection bias or other biases that may be present and how these are likely to impact results.*
Ethics oversight	*Identify the organization(s) that approved the study protocol.*

Note that full information on the approval of the study protocol must also be provided in the manuscript.

Field-specific reporting

Please select the one below that is the best fit for your research. If you are not sure, read the appropriate sections before making your selection.

☒ Life sciences ☐ Behavioural & social sciences ☐ Ecological, evolutionary & environmental sciences

For a reference copy of the document with all sections, see nature.com/documents/nr-reporting-summary-flat.pdf

Life sciences study design

All studies must disclose on these points even when the disclosure is negative.

Sample size	Sample sizes were estimated based on previous extensive experience in the laboratory with the EAE model.
Data exclusions	In the EAE model, mice without disease symptoms were excluded from the analyses.
Replication	In vivo EAE treatment experiments were performed twice (Fig. 5) or repeated with adapted strategies (Fig. 6, Fig. 7) as stated in the figure legends.
Randomization	In treatment EAEs, mice were randomly assigned to treatment or control groups. These mice were mixed within cages to minimize cage-specific effects.
Blinding	The experimenters were blinded for the genotype or the respective treatments.

Reporting for specific materials, systems and methods

We require information from authors about some types of materials, experimental systems and methods used in many studies. Here, indicate whether each material, system or method listed is relevant to your study. If you are not sure if a list item applies to your research, read the appropriate section before selecting a response.

Materials & experimental systems

n/a	Involved in the study
	[x] Antibodies
[x]	Eukaryotic cell lines
[x]	Palaeontology and archaeology
	[x] Animals and other organisms
[x]	Clinical data
[x]	Dual use research of concern
[x]	Plants

Methods

n/a	Involved in the study
[x]	ChIP-seq
	[x] Flow cytometry
[x]	MRI-based neuroimaging

Antibodies

Antibodies used

Biotinylated MR1-5-OP-RU, MR1-6-FP, CD1d-PBS-57 and empty CD1d monomers (2 mg/ml) were obtained from the National Institutes of Health (NIH), aliquoted and stored at –80°C. For tetramerization, monomers were incubated at a 4:1 molar ratio with fluorochrome labeled streptavidin (streptavidin-phycoerythrin, Thermo Fisher Scientific, S866 and streptavidin-BV421, BioLegend, 405225). Streptavidin was added stepwise at RT and tetramers were stored at 4°C. The working concentration varied between 1:300 and 1:800 in different tetramerization approaches. In order to minimize staining differences, newly prepared tetramers were tested against previous preparations on LN, spleen or liver cells.

Surface antigens were stained for 30 min at 4°C with respective fluorochrome-coupled antibodies from BioLegend against CD3ε (1:100, 145-2C11, 100306), CD4 (1:100, GK1.5, 100447, 100453), CD8 (1:200, 53-6.7, 100750, 100759), CD11b (1:100, M1/70, 101228), CD11c (1:100, N418, 117318), CD44 (1:200, IM7, 103012, 103032) CD45 (1:200, 30-F11, 103116, 103128), CD45R (1:100, RA3-6B2, 103248), CD69 (1:100, H1.2F3, 104512), CD317 (1:100, 927, 127016), Ly6G (1:100, 1A8, 127624), PD-1 (1:100, 29F.1A12, 135221), NK1.1 (1:100, PK136, 108708), TCR-β (1:100, H57-597, 109230), TCR-Vβ6 (1:100, RR4-7, 140006) or from BD Biosciences against CD11b (1:100, M1/70, 563553), CD19 (1:100, 1D3, 612971), CD45R (1:100, RA3-6B2, 612972), F4/80 (1:100, T45-2342, 565411), MHCII (1:100, M5/114.15.2, 563414), TCR-β (1:100, H57-597, 612821), TCR-Vβ8 (1:100, F23.1, 742378), TCR-γδ (1:100, GL3, 748989). Dead cells were stained using fixable viability stain 700 (BD Biosciences, 564997) for 20 min at 4°C.

For intracellular staining, fixation and permeabilization of single cell suspensions was performed according to the manufacturer's protocol (BioLegend, fixation buffer, 420801; intracellular staining perm wash buffer, 421002) and stained with labelled antibodies against IL-22 (1:50, 1H8PWSR, eBioscience, 46-7222-80), GM-CSF (1:50, MP1-22E9, BioLegend, 505412), IFN-γ (1:50, XMG1.2, BioLegend, 505838), IL-17A (1:50, TC11-18H10.1, BioLegend, 506916), AREG (1:00, R&D Systems, AF989,) for 60 min at 4°C. Unlabeled AREG antibody was conjugated to APC using the APC Conjugation Kit – Lightning-Link (abcam, ab201807) according to the manufacturer's instructions.

For intranuclear staining, cells endogenously expressed GFP were fixed with a 3% paraformaldehyde (PFA) solution for 60 min at RT to protect the GFP signal. Afterwards, the true-nuclear transcription factor buffer set (BioLegend, 424401) was used according to the manufacturer's instructions. Briefly, cells were fixed for 60 min at RT followed by two washing steps with permeabilization buffer and antibody staining against T-bet (1:50, 4B10, BioLegend, 644803) for 60 min at RT.

Validation

Antibody validation for the species is provided for all commercially available antibodies on the relevant manufacturer's website accessible via the catalogue numbers which are provided above.

Animals and other research organisms

Policy information about studies involving animals; ARRIVE guidelines recommended for reporting animal research, and Sex and Gender in Research

Laboratory animals

C57BL/6J wildtype (The Jackson Laboratory, Charles River), B6.129P2-Mr1tm1Gfn (Mr1–/–) (provided by Olivier Lantz, Paris, France), B6(Cg)-MaithiRorctm2Litt (RORγtGFP transgenic reporter mice) (provided by Olivier Lantz, Paris, France) and B6N.B6-Tg(Nr4a1-EGFP/cre)820Khog/J (Nur77GFP reporter mice, strain #018974 from the Jackson Laboratory) were housed and bred under specific pathogen-free conditions in the animal facility of the University Medical Centre Hamburg-Eppendorf. For experiments evaluating the effects of MAIT TCR modulation on EAE, 6–7-week-old female C57BL/6J wildtype mice were purchased from Charles River and acclimated to the new environment for at least 2 weeks before EAE induction. All mice were kept in a facility using a 12 h light/ 12 h dark cycle at temperatures of 20-25 °C with 40-70% humidity. Food and water were provided ad libitum. For experiments, we used adult mice (8–12 weeks old) from both sexes. For all experiments, littermates were used as control animals and mice from different experimental groups were mixed within cages to minimize cage-specific effects. Euthanasia was performed by using CO2 followed by cardiac puncture (PBS perfusion) or cervical dislocation.

Wild animals

N/A

Reporting on sex

Both sexes were used for most of the experiments. Treatment EAEs were performed with female mice as indicated in the figure legends.

Field-collected samples

N/A

Ethics oversight

All animal experimental procedures were in accordance to international and national animal welfare guidelines. Ethical approvals were obtained from the State Authority of Hamburg, Germany (approval no. 79/16 and 120/21).

Note that full information on the approval of the study protocol must also be provided in the manuscript.

Flow Cytometry

Plots

Confirm that:

- [x] The axis labels state the marker and fluorochrome used (e.g. CD4-FITC).
- [x] The axis scales are clearly visible. Include numbers along axes only for bottom left plot of group (a 'group' is an analysis of identical markers).
- [x] All plots are contour plots with outliers or pseudocolor plots.
- [x] A numerical value for number of cells or percentage (with statistics) is provided.

Methodology

Sample preparation	Cell isolation from mouse organs were performed as described in the Methods section. Single cell suspensions were incubated for 30 min at RT and 30 min at 4°C with MR1- or CD1d-tetramers at concentrations between 1:300 and 1:800. Surface antigens were stained for 30 min at 4°C with respective fluorochrome-coupled antibodies. For intracellular staining, fixation and permeabilization of single cell suspensions was performed according to the manufacturer's protocol (BioLegend, fixation buffer, 420801; intracellular staining perm wash buffer, 421002) and stained with labelled antibodies. For intranuclear staining, cells endogenously expressed GFP were fixed with a 3% paraformaldehyde (PFA) solution for 60 min at RT to protect the GFP signal. Afterwards, the true-nuclear transcription factor buffer set (BioLegend, 424401) was used according to the manufacturer's instructions. Briefly, cells were fixed for 60 min at RT followed by two washing steps with permeabilization buffer and antibody staining against T-bet (1:50, 4B10, BioLegend, 644803) for 60 min at RT.
Instrument	All samples were acquired on a BD FACS LSR II analyzer or FACSymphony A3 (BD Biosciences). Flow cytometry-based cell sorting was performed on a FACSAria III cell sorter (BD Biosciences).
Software	Flow cytometry data were collected with FACSDiva software version 9.1 (BD Biosciences) and analyzed with FlowJo analysis software version 10.8.
Cell population abundance	MAIT cell sorting purity was ≥ 95% and was checked by passing the post sort samples through the FACSAria III cell sorter.
Gating strategy	Gating strategy for MAIT (living CD45+CD11b–CD45R–TCR-β+MR1tetramer(5-OP-RU)+ cells) and non-MAIT T cells (living CD45+CD11b–CD45R–TCR-β+MR1tetramer(5-OP-RU)–CD44+ cells) are described in supplementary figure 1.

- [x] Tick this box to confirm that a figure exemplifying the gating strategy is provided in the Supplementary Information.
